# Possible macrophage activation syndrome following initiation of adalimumab in a patient with adult-onset still's disease

**DOI:** 10.11604/pamj.2014.17.94.3386

**Published:** 2014-02-07

**Authors:** Leila Souabni, Leila Dridi, Kawther Ben Abdelghani, Selma Kassab, Selma Chekili, Ahmed Laater, Leith Zakraoui

**Affiliations:** 1 Rheumatology Department, Mongi Slim Hospital, La Marsa, Tunis, Tunisia

**Keywords:** Adult-onset Still′s disease, macrophage activation syndrome, hemophagocytic syndrome, adalimumab

## Abstract

Macrophage activation syndrome (MAS) has been rarely reported in the course of adult-onset Still's disease (AOSD) and in the majority of cases, it was triggered by an infection. Here, we report, to our knowledge, the first case of MAS occurring after adalimumab treatment initiation and not triggered by an infection. A 26-yearold woman with classical features of AOSD developed persistent fever, severe bicytopenia associated with extreme hyperferritinemia, hyponatremia and abnormal liver function tow months after the initiation of adalimumab treatment. The diagnosis of MAS was made without histological proof. The patient was treated with methylprednisolone pulse therapy and her condition improved. During the disease course, extensive studies could not identify any viral infection or other known underlying etiology for the reactive MAS. The adalimumab was incriminated in this complication. Currently, the patient is in remission on tocilizumab and low-dose prednisolone.

## Introduction

Macrophage activation syndrome (MAS) is a serious, potentially fatal complication of rheumatic diseases which is most frequently in systemic juvenile idiopathic arthritis (sJIA) and its adult equivalent, AOSD. MAS may occur spontaneously as a complication of active underlying disease or may be triggered by an infection or toxic effect of a medication, including biologic agent [1]. We report, to our knowledge, the first case of AOSD presenting a MAS following the initiation of adalimumab treatment and not triggered by an infection.

## Patient and observation

A 26-year old female, was treated in rheumatology department for AOSD diagnosed in 2007. Initially, she was receiving 15 mg/ week of methotrexate 10 mg of prednisolone to control joint involvement. Our patient continued to have peripheral arthralgia with morning stiffness and a synovitis of the wrists, elbows and small joints of the hands.

In March 2010, a treatment with Anakinra 100mg/day was introduced. This treatment has allowed the amendment of systemic signs including fever, but it did not improve joint symptoms. Anakinra was substituted by a TNF alpha blocker: adalimumab 40mg/2weeks in September 2010 and methotrexate was increased to 20mg/week.

In November 2010, ten days after the last injection of adalimumab, she was hospitalized because of a persistant fever, joint pain and odynophagia. On examination, her temperature was 39.8°C, blood pressure 100/60 mm Hg and pulse rate 116/min. She was pale and asthenic. There was diffuse joint tenderness but not synovitis. She had a white blood cell count of 6700/mm^3^, microcytic anemia with hemoglobin rate of 7.7g/dl, erythrocyte sedimentation rate of 120 mm/hour and C-reactive protein of 225mg/dl. Liver and kidney blood tests were normal.

During hospitalization, the general condition rapidly deteriorated, fiver persisted ranging from 38 to 41° C and blood investigations showed exacerbation of anemia (Hb = 3.3g/dl), thrombocytopenia (100 000/mm^3^), elevated alanine aminotransferase (767 U/L; normal 0-40), aspartate aminotransferase (930 U/L; normal 10–35), elevated LDH (1183 U/L), a very high ferritin level (40955mg/l), hyponatremia (125mmol/l), hypocalcimea (1.52mmol/l) and high triglyceride level. Although there was no histological confirmation, diagnosis of secondary MAS was established, and the patient was transferred to the intensive care unit. Intravenous methylprednisolone was administered at 1g/day for 3 days relayed by oral route 2mg/kg/day for a period of one month then the degression of corticoid was started. Her fever subsided gradually, together with improvement and normalization of laboratory investigations results.

During the disease course, extensive studies could not identify any viral infection or other known underlying etiology for the reactive MAS. At this period, our patient was receiving corticosteroid 20mg /day, methotrexate 20 mg/week and adalimumab 40mg/2 week. Adalimumab was incriminated in this complication.

After a month of the MAS, it was decided to give tocilizumab. The treatment has allowed the maintaining of apyrexia and the regression of joints pain. There was improvement in all the biochemical parameters.

## Discussion

We have presented a case of AOSD complicated with MAS after the initiation of treatment by adalimumab. Adalimumab was prescribed for our patient because it has been effective in cases of refractory disease [2].

MAS, also referred to as secondary haemophagocytic syndrome (HS), is characterized by activation and uncontrolled proliferation of T lymphocytes and macrophages and the ingestion of cellular blood components and their precursors by these macrophages. Diagnosis of MAS involves 8 criteria of which 5 must be satisfied [3]. During the course of this case, only 4 of these criteria were achieved: fever, bicytopenia, hyperferritinemia and high triglyceride level. Even so, we retained the diagnosis of MAS for several reasons. A French study concluded that fever, cytopenia, ferritin > 1000mg/L and triglyceride> 2mmol/L, when associated, and as it was observed in our case, were the major criteria allowing an early diagnosis of MAS and justifying a specific treatment even in the absence of an histological proof [4]. Furthermore, it is very difficult to obtain the 5 criteria in early stage especially the histological one as well as splenomegaly [5].

Moreover, MAS and AOSD flare share several clinical features that may explain the difficulty in recognizing MAS complicating AOSD. Biological findings are more sensitive in evoking the diagnosis. High ferritin level, as it was found in our case, was extremely high (40955 mg/l) for a typical AOSD flare. In addition, our patient has thrombocytopenia and anemia aggravation that are poor prognostic factors and life tthreatening conditions. That's why, further investigation was judged to be not safe for the patient, and treatment was initiated soon.

The occurrence of MAS under TNF alpha blockers was reported in several cases. The first one was in 2003, it was about a child with systemic onset juvenile rheumatoid arthritis who developed MAS after initiation of etanercept therapy [6]. The etanercept was also incriminated in the occurrence of MAS in a 42-year-old woman with a history of rheumatoid arthtritis [7]. Infliximab was implicated in the occurrence of MAS prescribed for a 37-year-old man with fistulated Crohn's disease unresponsive to azathioprine [8]. Larroche et al reported 2 cases of men with ankylosing spondylitis and spondylarthropathy associated with inflammatory bowel disease who have developped a MAS under infliximab [9].

Concerning adalimumab, the first case of MAS in a patient treated by adalimumab was reported, in our knowledge, in 2010 by Molto et al, it was a man of 60years old with rheumatoid arthritis, he developed visceral leishmaniasis and MAS one year after the initiation of the treatment [5]. The MAS was imputed in this case to the visceral leishmaniasis, and adalimumab was considered as a predisposing cause of this infection.

Only two previous cases of MAS in patients with AOSD during adalimumab therapy have been documented in the literature [2, 10]. In the tow cases, the disease course on adalimumab was complicated by the occurrence of disseminated histoplasmosis with MAS.

In our case extensive investigations could not identify any infectious etiology for the MAS which make the responsibility of adalimumab very likely. This complication occurred 2 months after treatment initiation and that is a timeline argument. Our patient was also receiving methotrexate. The responsibility of methotrexate in our case is very unlikely because it was prescribed during 4 years ago without any side effect and its reintroduction after the MAS was safe.

## Conclusion

Our patient serves to heighten awareness of MAS as a potential complication of AOSD when adalimumab is used in treatment and to insist on the importance of early reorganization of this life-threatening complication even in the absence of histological proof.
